# Possible Immunomodulating Effect of Retinol on Cytokines Secretion in Patients with Recurrent Furunculosis

**DOI:** 10.1007/s00005-017-0483-5

**Published:** 2017-07-20

**Authors:** Danuta Nowicka, Ewelina Grywalska, Anna Hymos, Michał Mielnik, Jacek Roliński

**Affiliations:** 1 Department of Dermatology, Venereology and Allergology, Medical University of Wrocław, Chalubinskiego 1, 50-368 Wroclaw, Poland; 2 Department of Clinical Immunology and Immunotherapy, Medical University of Lublin, Lublin, Poland; 3 Department of Otolaryngology and Laryngeal Oncology, Medical University of Lublin, Lublin, Poland

**Keywords:** Retinol, Recurrent furunculosis, Immune response, Skin infection, In vitro study

## Abstract

Recurrent furunculosis is an infection of hair follicles which results in formation of abscesses. Previous studies showed that the pathogenesis of the disease may include an immune-mediated component as the proliferative response of peripheral blood lymphocytes to staphylococcal antigen is depressed. The aim of our study was to evaluate cytokines concentration in the plasma of patients with recurrent furunculosis and to determine whether retinol affects the secretion of those cytokines in patients with recurrent furunculosis and healthy subjects. Blood samples were taken from 15 patients with recurrent furunculosis and 15 age-matched healthy subjects. A quantitative determination of selected cytokines (IL-17, 13, 2, 10, 4, IFN-γ, TNF-α) was performed in the plasma at baseline and after 72-h culture of peripheral blood mononuclear cells with and without retinol in both groups. In the plasma of patients with recurrent furunculosis, concentration of IL-10, 2, and TNF-α was significantly higher, whereas IL-13 significantly lower when compared with healthy subjects. After retinol stimulation, the concentration of IL-17 and IFN-γ increased significantly in both groups. Secretion of anti-inflammatory cytokines, especially IL-10 (*p* < 0.002) and 13 (*p* < 0.01), achieved lower levels in recurrent furunculosis samples than in those of healthy controls. Network of cytokines differs in patients with recurrent furunculosis from healthy subjects. Retinol stimulation affects secretion of both pro-inflammatory and anti-inflammatory cytokines. Further studies are recommended for better understanding the pathomechanism of recurrent furunculosis and potential clinical use of retinol in patients affected by recurrent furunculosis.

## Introduction

Recurrent furunculosis is an infection of hair follicles which results in formation of abscesses accumulating pus and necrotic tissue. Furuncles are common on the hair-bearing parts of the skin. Most cases are associated with *Staphylococcus aureus* (*S. aureus*) infection, but other bacteria may also contribute to the development of this disease (Dahl 1987; Ibler and Kromann 2014). In cases associated with low efficiency of antibacterial and symptomatic therapy, the disease typically becomes chronic or recurrent. Three or more relapses within a year period are classified as recurrent furunculosis (Ibler and Kromann 2014). Colonization of *S. aureus* in the anterior nares carriage plays an important role in the etiology of chronic or recurrent furunculosis (Demos et al. 2012; El-Gilany and Fathy 2009; Ibler and Kromann 2014). It is well documented that the ability of peripheral blood lymphocytes to respond to various stimuli including staphylococcal antigen is significantly depressed in patients with furunculosis as compared with healthy controls and may be associated with immune suppression (Hamaliaka and Novikova 2010; Tsuda 1977). The association between cellular and humoral immunity in furunculosis should be further studied.

Retinol is the most active alcohol form of vitamin A. It plays an important role in cell growth and differentiation. It is stored mainly in the liver and carried to the peripheral tissues by retinol binding protein (RBP). Impaired expression of RBP may alter inflammatory mechanisms in adipose and vascular tissues as well as impair immune responses (Zabetian-Targhi et al. 2015). In the skin, retinol regulates proliferation of fibroblasts, and thus plays a crucial role in the synthesis of collagen and elastic fibers. It normalizes the process of dead cell shedding from the surface of stratum corneum through its effect on proteolytic enzymes and regulates keratinization of hair follicle, and therefore exerts anti-comedogenic effect. It also induces angiogenesis and hyperpigmentation after exposure to the sun. Retinol may exert anti-inflammatory action by the effect on transformation of arachidonic acid (Hope et al. 1990). It is known and widely used factor in the prevention and banishing the signs of skin aging, especially induced by the sun (Shao et al. 2017). It is recommended in the treatment of severe acne and seborrhea. It can be used as an adjunct therapy in the combination treatment for psoriasis, lichen planus, lichen ruber follicularis and other diseases with impaired keratinization of the epidermis.

The aim of our in vitro study was to evaluate concentration of cytokines in the plasma of patients with recurrent furunculosis. Additionally, we aimed to determine whether retinol affects the secretion of cytokines by peripheral blood mononuclear cells (PBMCs) obtained from patients with recurrent furunculosis and healthy subjects.

## Materials and Methods

### Characteristics of Patients and Healthy Volunteers

The study included peripheral blood samples obtained from 15 patients with recurrent furunculosis (eight women and seven men) in remission phase. Blood samples were collected after obtaining written informed consent. The age of the study patients ranged 25–45 years; mean 34 ± 9 years. The control group included 15 age-matched healthy subjects (eight women and seven men). Neither the patients nor the controls used immunomodulating agents or hormonal preparations, presented signs of other than furuncle skin infection within at least 3 months prior to the study, underwent blood transfusion, or presented with autoimmune condition or allergy. Additionally, none of the patients and controls had a history of oncological therapy or prior treatment for tuberculosis or any other chronic condition that could be associated with impaired cellular or humoral immunity.

The study was approved by the Ethics Committee of the Medical University of Lublin (Decision no. KE-0163/291/2012).

### Examined Material

Peripheral blood from the basilic vein of patients and healthy controls was collected into EDTA-treated tubes (15 ml; aspiration and vacuum systems Sarstedt, Germany). Immediately after collection, the samples were used for isolation of mononuclear cells for in vitro tests and plasma collection.

### Isolation of Mononuclear Cells and Plasma

Ten ml of peripheral blood was diluted with 0.9% buffered saline (PBS) without calcium (Ca^2+^) and magnesium (Mg^2+^) (Biochrome AG, Germany) in 1:1 ratio. The diluted material was built up with 3 ml of Gradisol L (specific gravity 1.077 g/ml; Aqua Medica, Poland), and centrifuged in a density gradient at 700×*g* for 20 min. The obtained fraction of PBMCs was collected with Pasteur pipettes and washed twice in PBS without Ca^2+^ and Mg^2+^ for 5 min. Subsequently, the cells were suspended in 1 ml of PBS without Ca^2+^ and Mg^2+^, and either counted in the Neubauer chamber or tested for viability with trypan blue solution (0.4% Trypan Blue Solution, Sigma Aldrich, Germany). Viability below 95% disqualified the cells from further analyses.

Plasma was obtained from the remaining 5 ml of blood samples, aliquoted, and stored at −80 °C for enzyme-linked immunosorbent assay (ELISA) test.

### Cell Culture

After isolation, the cells were cultured for 72 h in the complete culture medium RPMI 1640 (PAA Laboratories, Austria) with 10% human albumin (Baxter, USA) and antibiotics: penicillin (100 IU/ml), streptomycin (50 μg/ml) and neomycin (100 μg/ml) (Sigma Aldrich, Germany) in an amount of 1 ml. Cells were cultured at 37 °C in 5% CO_2_ atmosphere. PBMCs obtained from each individual were cultured with retinol (in the dose of 100 μL per sample) and without retinol.

### Cytokine Assessment

For the study, we have chosen the following cytokines: intereleukin (IL)-17, interferon (IFN)-γ, IL-4, tumor necrosis factor (TNF)-α, IL-13, 2, 10 as they play an important role in the inflammatory process. Commercial ELISA kits, purchased from R&D Systems (USA) for a quantitative determination of selected cytokines in human plasma and cell culture supernatants were used in accordance with the manufacturer’s recommendations. The ELISA Reader Victor TM3 (PerkinElmer, USA) was used for measurements.

### Statistical Analysis

Statistical analysis of the results was conducted using Statistica 10 (StatSoft, Tulsa, OK, USA). Deviation from normality was evaluated by the Kolmogorov–Smirnov test. Differences between groups were assessed using the Wilcoxon test. Data were expressed as the mean value ± SD, minimum and maximum. A value *p* < 0.05 was considered statistically significant.

## Results

### Assessment of Cytokines in the Plasma of Patients with Recurrent Furunculosis

In the plasma, all cytokines of subjects with recurrent furunculosis had higher concentration than those of healthy individuals, with the exception of IL-13, which concentration was significantly lower compared with healthy controls (*p* < 0.05). Concentration of IL-4 and ten in patients was higher than in healthy subjects, but only IL-10 concentration reached the level of statistical significance (*p* < 0.01). Other cytokines showed higher concentration in patients with recurrent furunculosis; however, differences were significant only in the case of IL-2 (*p* < 0.02) and TNF-α (*p* < 0.00001; Table 1).

**Table 1:** The assessment of selected cytokines in the plasma of patients with recurrent furunculosis and health controls

	Mean	SD	Min.	Max.	*p* value
IL-17 (pg/ml) study group	4.6136	1.1042	2.1434	8.2875	NS
IL-17 (pg/ml) control group	4.5563	1.2446	2.2772	6.4924	
IFN-γ (pg/ml) study group	3.3805	1.8858	0.0864	8.2165	NS
IFN-γ (pg/ml) control group	2.3064	1.8257	0.1144	6.0232	
IL-4 (pg/ml) study group	6.4956	3.0530	3.2342	23.0506	NS
IL-4 (pg/ml) control group	6.1065	1.0094	4.7023	8.8665	
TNF-α (pg/ml) study group	0.9424	0.7588	0.3708	4.7491	0.000004
TNF-α (pg/ml) control group	0.3923	0.1386	0.1602	0.7221	
IL-13 (pg/ml) study group	17.6211	4.9879	12.4988	38.9274	0.048046
IL-13 (pg/ml) control group	19.0769	5.2646	13.5192	35.3805	
IL-2 (pg/ml) study group	8.5305	2.9669	4.2768	18.1398	0.016460
IL-2 (pg/ml) control group	6.7100	1.3443	4.7755	10.1191	
IL-10 (pg/ml) study group	7.5772	4.6268	1.8709	32.5254	0.004243
IL-10 (pg/ml) control group	5.1872	1.8707	3.4646	10.5293	

*NS* not significant

### Assessment of Cytokines in the Supernatants After 72-h Culture of PBMCs with Retinol

**Fig. 1:**
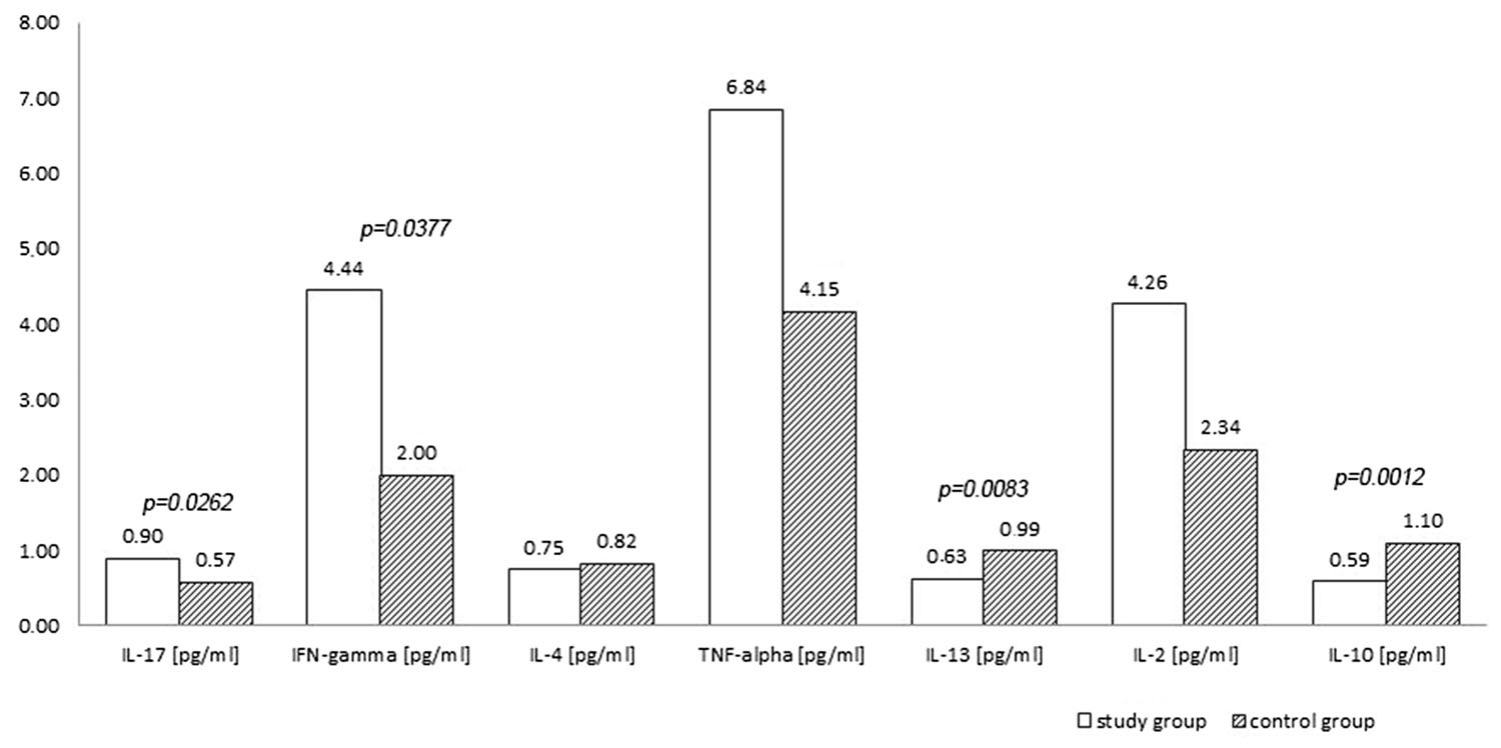
Cytokines concentration in the supernatants after 72-h culture of peripheral blood mononuclear cells with retinol (study group vs. controls)

Stimulation of retinol showed differences in cytokine production by PBMCs derived from patients and healthy controls. Production of pro-inflammatory cytokines after stimulation by retinol was higher in patients; the statistical significance was observed in case of IL-17 (*p* < 0.03) and IFN-γ (*p* < 0.04); the plasma concentration of IL-2 and TNF-α, although higher showed no statistically significant differences. In contrast, anti-inflammatory cytokines after stimulation by retinol were produced in smaller amounts in patients than in controls, which was especially visible in case of IL-10 (*p* < 0.002) and 13 (*p* < 0.01). The difference in IL-4 concentration was insignificant (Fig. 1).

### Assessment of Cytokines in the Supernatants After 72-h Culture of PBMCs with Retinol and Without Retinol

**Fig. 2:**
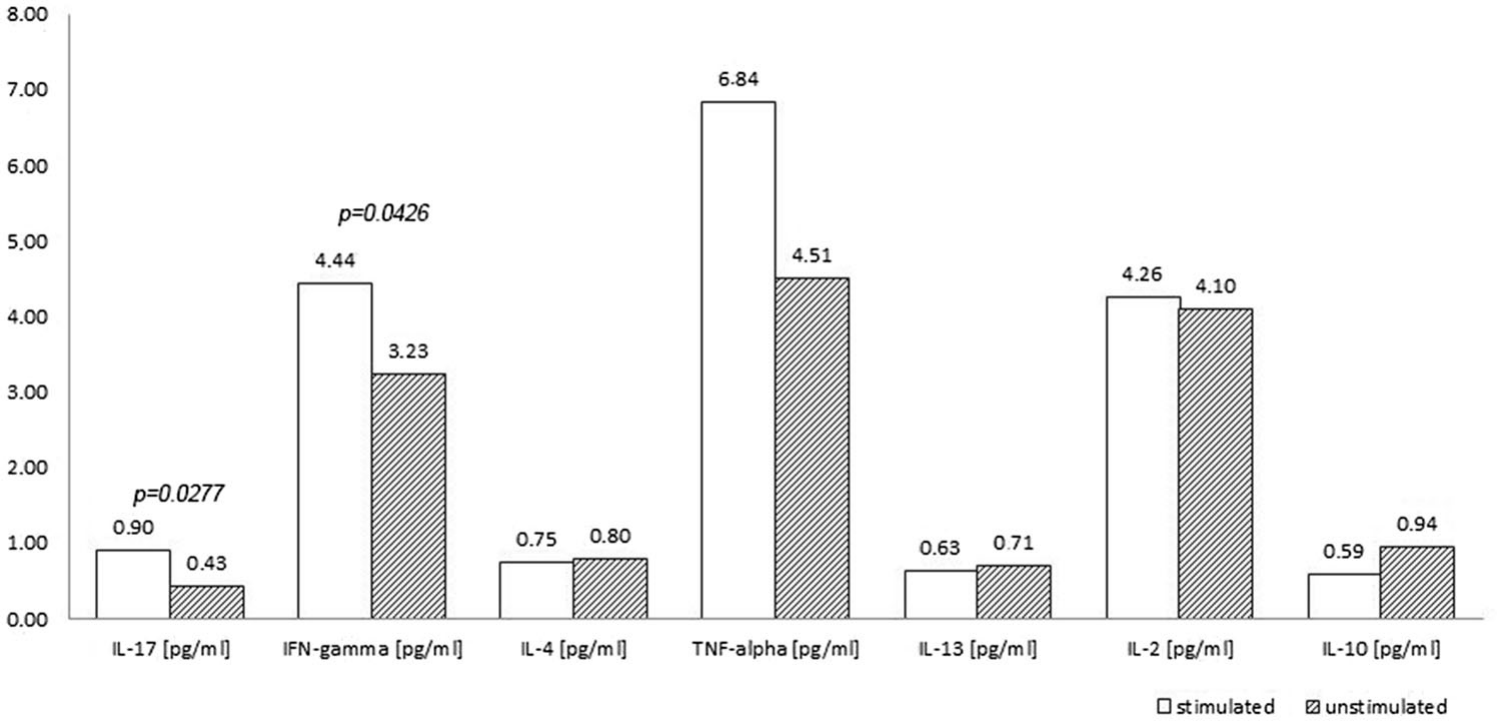
The assessment of cytokines in the supernatants after 72-h culture of peripheral blood mononuclear cells with retinol and without retinol stimulation in the group of patients

The measurement of cytokine concentration in the supernatant culture of unstimulated PBMCs of patients, revealed a reduction in the concentration of all three tested anti-inflammatory cytokines, and, among the pro-inflammatory cytokines, IL-2; however, the obtained reduction was not statistically significant. Due to the stimulation of PBMCs by retinol, concentration of pro-inflammatory cytokines such as IL-17 (*p* < 0.03) and IFN-γ (*p* < 0.05) increased significantly. Other differences were not statistically significant (Fig. 2).

In healthy individuals, both concentration of IL-17 in the supernatant and IFN-γ increased significantly (*p* < 0.02 and *p* < 0.03, respectively) in response to retinol stimulation. The concentration of anti-inflammatory cytokines showed a significant increase in the level of IL-10 (*p* < 0.04). Other differences were not statistically significant (Table 2).

**Table 2:** The assessment of cytokines in the supernatants after 72-h culture of peripheral blood mononuclear cells with retinol and without retinol in the group of healthy controls

	Mean	SD	Min.	Max.	*p* value
IL-17 (pg/ml) stimulated	0.5727	0.2281	0.2200	0.9900	0.016292
IL-17 (pg/ml) unstimulated	0.1252	0.1397	0.0137	0.5318	
IFN-γ (pg/ml) stimulated	2.0007	1.2000	0.6000	5.6200	0.025385
IFN-γ (pg/ml) unstimulated	0.6482	0.8757	0.0974	2.8476	
IL-4 (pg/ml) stimulated	0.8187	0.2440	0.5000	1.2700	NS
IL-4 (pg/ml) unstimulated	0.7983	0.2193	0.4791	1.7638	
TNF-α (pg/ml) stimulated	6.8367	6.8088	0.0600	28.4400	NS
TNF-α (pg/ml) unstimulated	6.6482	6.5477	0.0387	25.7496	
IL-13 (pg/ml) stimulated	0.9893	0.3487	0.4300	1.5500	NS
IL-13 (pg/ml) unstimulated	1.0038	0.4183	0.3675	1.3426	
IL-2 (pg/ml) stimulated	2.3380	1.0798	0.8500	4.7300	NS
IL-2 (pg/ml) unstimulated	2.4037	0.9987	0.7965	5.0001	
IL-10 (pg/ml) stimulated	1.0967	0.3151	0.6300	1.5300	0.036102
IL-10 (pg/ml) unstimulated	0.6282	0.2682	0.1839	1.9827	

*NS* not significant

## Discussion

The interaction between cells of the immune system is possible through cytokines produced by them. The concentration of those cytokines in the plasma is more and more often used for description of various pathological processes. Constellation of appropriate cytokines is being discovered not only in inflammatory, allergic, and cancer processes, but also in stress and aging of the body (Minciullo et al. 2016). In patients with recurrent furunculosis, concentration of cytokines in the plasma exerted increased pro- and anti-inflammatory activity; however, significantly higher concentrations occurred only in case of TNF-α and IL-2 among pro-inflammatory cytokines and IL-10 among anti-inflammatory cytokines. The rise of TNF-α in the plasma of patients in comparison with healthy volunteers draws particular attention. The increased production of this cytokine in patients is rooted in the stimulation of granulocyte colony-stimulating factor to production of IL-17, granulocyte macrophage colony-stimulating factor (GM-CSF), IL-1, 3, 4, TNF-α and IFN-γ. This result depends on the activity of all human body cells—not only mononuclear cells studied in the present paper. IL-10 exerts inhibiting impact on pro-inflammatory cytokines. The analysis of its concentration in the plasma revealed that IFN-γ to IL-10 ratio is similar in patients suffering from recurrent furunculosis (0.446) to healthy people (0.4446). Perhaps the concentration of this cytokine determines to a large extent the inhibition of the disease and only periodic occurrence of skin lesions. Surprisingly, IL-13 presented lower concentration in the plasma in patients with recurrent furunculosis in comparison with healthy people. IL-13 belongs to anti-inflammatory cytokines. It is secreted mostly by activated Th2 cells, but IL-13 receptors are expressed on many types of cells, therefore, it can influence many cells (Hershey 2003). It can stimulate production and activation of transforming growth factor β, fibroblast proliferation, differentiation of myofibroblasts, and expression of matrix metalloproteinases (Fuschiotti et al. 2013; Kendall and Feghali-Bostwick 2014) L-13 is a cause of the suppressive effect on inflammation in the processes of bacterial infections. Perhaps, the concentration of this cytokine is crucial for patients with recurrent furunculosis. Supposedly, this may be associated with the impaired expression of integrins, which are elevated by IL-13 in monocytes and macrophages of patients with recurrent furunculosis (de Vries 1998).

In vitro, PBMCs of patients with recurrent furunculosis cultured with retinol secreted more IL-17 and IFN-γ than those without stimulation by retinol. Mononuclear cells of healthy people showed similar properties, but the amount of cytokines obtained from cultures of patients with recurrent furunculosis was significantly higher than those of healthy people. IL-17 mediates inflammatory reaction in various types of tissue. It is secreted by lymphocytes Th1 and Th2 as well as by monocytes/macrophages and affects fibroblasts which induce production of macrophage inflammatory protein 2, nitric oxide, and prostaglandins. It also influences maturation of dendritic cells, recruitment of monocytes/macrophages, and accumulation of neutrophils necessary to create abscesses. It was revealed in the murine model that administration of an anti-body against IL-17 prevented abscess formation (Chung et al. 2003). In certain skin diseases, IL-17 increases the expression of adhesion molecules and directly or indirectly modulates IFN-γ- and IL-4-induced activation of keratinocytes leading to intensified production of GM-CSF (Albanesi et al. 2000). Thus, it seems that readiness for abscess formation in the skin of patients with recurrent furunculosis is higher than those of healthy subjects.

In 3-day cultures of mononuclear cells, the impact of retinol on secretion of anti-inflammatory cytokines in patients with recurrent furunculosis differed from those in healthy volunteers. Significant differences in production of IL-13 between both groups were not revealed, and thus it should be concluded that the stimulation did not affect the concentration of IL-13. However, the comparison of cytokines concentration in the supernatant after stimulation of mononuclear cells between patients with recurrent furunculosis and healthy subjects showed significantly lower levels in ill than in healthy subjects (*p* = 0.008). This relation may indicate retinol-dependent impaired mechanism of IL-13 production by mononuclear cells of patients with recurrent furunculosis.

The production of IL-10 by mononuclear cells presented itself differently. Cells of patients with recurrent furunculosis produced insignificantly less in comparison with unstimulated cells, while mononuclear cells of healthy subjects presented significant increase in production of this cytokine after retinol stimulation (*p* = 0.001). One can observe here different effect of retinol on mononuclear cells of patients with recurrent furunculosis, in whom retinol did not increase production of anti-inflammatory cytokines.

In mice and rats, vitamin A reduces the production of IFN-γ, but it increases the production of IL-4, 5, 10a, and namely production of anti-inflammatory cytokines (Long and Santos 1999). The drop in vitamin A increases IFN-γ and IL-12, but not IL-4 and 10 (Cantorna et al. 1994; Long and Santos 1999). These results are in line with Jason et al. (2002) outcomes who examined IL-10 to TNF-α concentration ratio in African children in relation to HIV infection status, the presence of *Mycobacterium bovis* bacillus Calmette-Guerin vaccine scarring, and vitamin A level in the plasma (reduced <10 μg/dL or normal ≥20 μg/dL). Their results indicate that proper vitamin A supplementation was associated with an increase in IL-10 concentration and/or decrease in TNF-α, while vitamin A deficiency was associated with the opposite results (Jason et al. 2002).

In our experiment, only increase in production of IL-10 after stimulation of monocytes/macrophages in the control group of healthy volunteers is in line with previous studies. The lack of experiments which included concentration of studied herein cytokines in relation to retinol stimulation does not allow us for further comparisons. However, it seems that depending on the disease process being the source of the studies cells, the response may not be the same. For this reason as well as for better understanding of the pathomechanism of many diseases, network of cytokines should be further studied.

It must be noted that inflammatory mechanisms are extremely complex, which means that not only cultured mononuclear cells with and without retinol contribute to the expression of pro- and inflammatory cytokines. Examination of only this particular aspect of pathomechanism of recurrent furunculosis is a limitation of this study, as it does not present a wide picture of the disease; however, we hope it can contribute to understanding of mechanisms underlying immune- and inflammatory related diseases.

On the basis of our research, we conclude that:

1. The plasma of patients with recurrent furunculosis differed from the healthy subjects in terms of significantly higher concentration of pro-inflammatory cytokines such as TNF-α and IL-2 and anti-inflammatory cytokines such as IL-10.
2. It is noteworthy that in patients with recurrent furunculosis, IL-13 concentration in the plasma was significantly lower and its production by monocytes/macrophages after stimulation by retinol in 3-day culture was reduced when compared with the group of healthy controls.
3. Retinol stimulation exerts elevated production of IL-17 and IFN-γ, but only a small increase in production of IL-13 and 10 in patients with recurrent furunculosis when compared with healthy subjects.

## Abbreviations

IL: Interleukin
GM-CSF: Granulocyte macrophage colony-stimulating factor
HIV: Human immunodeficiency virus
IFN: Interferon
PBMCs: Peripheral blood mononuclear cells
PBS: Phosphate buffered saline
TNF: Tumor necrosis factor
